# A pilot study examining periodontally healthy middle-aged humans and monkeys display different levels of alveolar bone resorption, gingival inflammatory infiltrate, and salivary microbiota profile

**DOI:** 10.1371/journal.pone.0311282

**Published:** 2024-10-16

**Authors:** Bingpeng Lin, Janak L. Pathak, Hongbin Gao, Zijun Zhou, Hooi-Leng Ser, Lihong Wu, Learn-Han Lee, Lijing Wang, Jianming Chen, Mei Zhong

**Affiliations:** 1 Department of Orthodontics, Affiliated Stomatology Hospital of Guangzhou Medical University, Guangzhou, China; 2 Guangzhou Key Laboratory of Basic and Applied Research of Oral Regenerative Medicine, Affiliated Stomatology Hospital of Guangzhou Medical University, Guangzhou, China; 3 Guangdong Laboratory Animals Monitoring Institute, Key Laboratory of Guangdong Laboratory Animals, Guangzhou, China; 4 School of Life Sciences and Biopharmaceutics, Guangdong Pharmaceutical University, Guangzhou, China; 5 Novel Bacteria and Drug Discovery (NBDD) Research Group, Microbiome and Bioresource Research Strength (MBRS), Jeffrey Cheah School of Medicine and Health Sciences, Monash University Malaysia, Subang Jaya, Malaysia; 6 Microbiome Research Group, Research Centre for Life Science and Healthcare, Nottingham Ningbo China Beacons of Excellence Research and Innovation Institute(CBI), University of Nottingham Ningbo China, Ningbo, China; University of Pennsylvania, UNITED STATES OF AMERICA

## Abstract

**Background:**

Monkeys are an appropriate model for periodontal research owing to their similar dental anatomy and physiology unlike humans. Extensive literature exists on pathological periodontitis in monkeys and humans, although concerns regarding whether healthy middle-aged monkeys and humans display the same periodontal and oral microbial status remains unclear.

**Aims and objectives:**

The current study aimed to compare alveolar bone resorption, gingival inflammatory infiltrate, and salivary microbiota profile in periodontally healthy middle-aged humans and monkeys.

**Methods:**

CBCT examination and histological analysis were performed to compare the periodontal status in middle-aged healthy humans and monkeys. Oral saliva16S rRNA sequencing was performed to analyze the oral microbial profile.

**Results:**

The alveolar resorption was compared between humans and monkeys, to determine the periodontal health. The percentage attachment of attachment loss was more around the posteriors teeth in humans when compared to monkeys (p<0.05). The degree of gingival inflammation was analyzed in both the groups, the expression of CD 34,45was higher in humans. 16S rRNA analysis demonstrated less diversity of salivary microorganisms in humans than in monkeys. The relative abundance of *Aggregatibacter*, *Haemophilus*, *Gemella*, and *Porphyromonas* at the genus level was significantly less in humans than in monkeys (p(<0.05).

**Conclusion:**

The periodontally healthy middle-aged humans and monkeys display different alveolar bone resorption and gingival inflammatory infiltrate levels. Furthermore, the salivary microbiota profile showed distinctly different oral microbiomes in these two primates. Our results suggest that the difference in alveolar bone status and gingival inflammatory infiltrate in healthy humans and monkeys might be associated with the diversity of the oral microbiome.

## Introduction

Periodontal disease often presents as chronic infections in the oral cavity of adults, which can be characterized by the loss of supporting structures around the affected teeth. The prevalence of periodontitis differs in various age groups, for incidence gingivitis is said to be most common periodontal disease among adolescence in China. The most common occurrence of periodontitis can be seen in age group 40–60 yrs. Eke et al stated that in US the prevalence of periodontitis can be found in early 30 s, which can be attributed to various local and systemic factors. [1–5]. There are many etiological factors of periodontitis which include local and systemic factorsleading to loss of alveolar bone up to 3mm and destruction of supporting structures [6–8]. Previous research data has demonstrated that periodontitis has been linked with increased systemic levels of cytokines, such as interleukin-1α,1β, interleukin-6 (IL-6), C-reactive protein (CRP), TNF tumor necrosis factor-α and interferon-γ in serum or plasma of periodontal compromised individuals. Systemic level changes of cytokines in periodontitis patients indicate that inflammation plays a role in the etiologic mechanisms of systemic inflammatory diseases, and inflammation drives the pathogenesis of periodontitis. The identification of Periodontal risk using biomarkers in severe inflammatory condition such as, N-terminal pro b-type natriuretic peptide (NT-proBNP) has been identified. BNP is a 32 amino acid peptide, which is increased in cardiovascular diseases. The inactive biological hormone is called NT-Pro BNP. There are evident studies regarding the association of periodontitis and NTPro BNP levels. After post scaling and root planning the levels of NT pro BNP were decreased in a study done by Fazal et al. The reasons may be upon the removal of local factors,the inflammatory response may be reduced Hence the mediators which stimulate the production of (NT-proBNP)–acute phase protein may be reduced. Also the serum levels should be evaluated frequently in periodontitis due to time required for stabilization in blood stream which may be 3–6 months. The other cause may be bacteremia which is seen immediately after scaling and root planning hence there may be rise of serum NT-pro BNP levels for brief time. However evident studies have been still lacking regarding association of periodontitis and increased NT-pro BNP [9].

The TGF factor β1 is a multifactorial cytokine which plays a key role in cellular process such as differentiation, apoptosis growth immune reactions and angiogenesis. The inflammatory cells are known to synthesizeTGF β1, it increases expression of Osteoprotegerin which is known to have inhibitory role in destruction of alveolar bone during periodontal diseases. The presence of TGF β1 in GCF leads to epithelial cell destruction induces apoptosis hence it can be used as target for monitoring progression of periodontitis. TGF β1 promotes growth, cell differentiation and matrix production with increased collagen formation, which is responsible for progression in periodontitis. The gene polymorphisms ofTGF β1 are responsible factors for the susceptibility of periodontitis. The TGF β1 also plays a key role in tooth supporting tissue by prompt remodeling of connective tissue and angiogenesis. The relationship regarding and risk of oxidative stress evolution that could improve the quality of life in periodontitis patients and periodontal cells also have been important. The neutrophils are said to be the most important inflammatory cells in periodontal tissue and gingival sulcus. They are periodontal sources of reactive oxygen species ROS in periodontitis. They are predominant resources of reactive oxygen species. The ROS related degradants which may be enzymatic and non enzymatic are candidates to assess antioxidant activity, and oxidative stress related events in pathological process of periodontitis. Some of the local markers such as MDA (Malondialdehyde), hydrogen peroxideand oxidative DNA damage has been reported in periodontitis. The elevated levels of biomarkers, ROS induced tissue damage and elevated levels of antioxidant enzymes are related to oxidative stress in inflamed periodontaltissue and gingival fluid [10].

Zhao et al. investigated the prevalence and severity of alveolar bone loss in the middle-aged (40–59 years) Chinese population [2]. They found a higher degree of alveolar bone loss in females than in males. Furthermore, the evidence regarding the association of periodontitis in middle-aged adults with the development of chronic diseases, such as type 2 diabetes, atherosclerosis, and rheumatoid arthritis is not much documented [11–13]. Therefore, the periodontal health status in the middle-aged population is directed more towards theoral microbiome status. The oral microbiome is one of the most complex and dynamic microbial communities in the human body. The salivary microbiome comprises aerobic and anaerobic bacteria. Takeshita et al stated that the periodontal health is dependent on these variants which reflect the oral environment and are the causative factors for periodontitis (Takeshita et al., 2014), [14]. They also analyzed the salivary microbiome from 200 human subjects,and compared the results with periodontal health conditions, and found the predominance of *Neisseria* with healthy periodontal conditions [15]. Moreover, the relative abundance of predominant bacteria in the saliva is significantly associated with oral health- conditions. However, the mechanisms behind the differences in the salivary microbiome affecting periodontal health is not well understood. Salivary *Spirochaetes* is found more in adults and less in elders which could confer the reasons for periodontal diseases in chinese population [16]. The oral microbiome represents a balanced dynamic ecosystem. The16S ribosomal ribonucleic acid (rRNA) gene sequencing is widely used technique for determining the prokaryotic communities’ diversity, structure and composition associated with oral health and disease states. Moreover, this technology is also widely employed to study alterations in the mouth microbiome associated with the development and progression of various systemic pathologies, such as diabetes, cardiovascular diseases, rheumatoid arthritis, Alzheimer’s disease and respiratory disorders, as well as adverse outcomes of conditions such as pregnancy and oral diseases such as periodontitis [17]. From years the research has been focused on relationship of several taxa and periodontitis. Although the oral microbiome with severe periodontitis has particularly focused on red complex bacteria. The reason for dysbiosis and healthy patients is still unclear. There are almost 30 oral taxa which are assigned to periodontal health. There are still many new taxa which are unidentified. The metabolic activity in the oral habitat and host response to microbial production could be distinguishing factor between oral health and dysbiosis. The ultimate evaluation of proteomic and metagenomic analysis in future would yield better results on identification of new taxa. Furthermore, the relative abundancesof predominant bacteria is known to vary with theaging process [18, 19]. Hence in the current study we aimed at investigating the relationship between salivary microbial profile and periodontal health in middle aged population. Experimental models resembling the anatomical and pathophysiological conditions of humans are essential for understanding the etiopathogenesis and changes in periodontal status under different pathophysiological conditions [20, 21]. Non-human primates represent animal models that closely resemble humans in dental anatomy and physiology. Periodontal diseases with clinical symptoms and host immune status in non-human primates can be easily compared withhumans [22–24]. Extensive literature exists on naturally occurring or experimental periodontitis in monkeys and humans [18]. To the best of our knowledge, there is no available literaturecomparing the periodontal status and oral microbiota profile of periodontally healthy middle-aged humans and monkeys. Thus, the purpose of the present study was to analyze the periodontal status using CBCT and salivary microbiome profile in healthy middle-aged humans. Also we aimed in correlating the periodontal status and salivary profile in monkeys and compare them in these two primates.

## Methods

### Human samples

**The inclusion criteria was** Patients (n = 5, female) were recruited in this study with age 40 to 55 yrs The subjects with gingivitis, probing depths (PD) > 3 mm, and diagnosed periodontal disease were all included.

### Exclusion criteria

1)The patients who had no uncontrolled medical conditions, with normal blood chemistries 2) Those who are not on corticosteroids or regular antibiotics during the last three months 3) Those who are not pregnant,nor on immunosuppressive drugs 4) The subjects with gingivitis, probing depths (PD) < 3 mm, history of smoking, and periodontal disease were all excluded [25]. The gingiva tissue samples from the middle-aged woman were collected from a corresponding anatomical site during premolars extractions for orthodontics or surgical removal of third molars. All human participants signed the informed consent before undergoing the procedures for cone-beam computed tomography (CBCT) and for collection of tissue samples. All the experimental procedures were carried according to the declarartion of helisinki revised in 2003. The study was approved byInstitution Ethics Committee of Affiliated Stomatology Hospital of Guangzhou Medical University.

### Animal samples

For this study, inclusion criteria was four female monkeys (*Macaca fascicularis*, 11–15 years old, bodyweight 2.6–3.6 kg, Guangdong Chunsheng Biotechnology Co., Ltd) with clinical features BOP < 1, probing depths < 3 Each animal was offered a measured amount of homemade pellet feed, mainly papaya, rice, sugar, soybeans and wheat. Fresh potable drinking water was provided ad libitum. Before sample preparation, subjects were anesthetized with intramuscular administration of ketamine (15mg/kg). CBCT(Cone-beam computed tomography) and gingival tissue biopsies were performed similarly to a human clinical study. The health of each animal was monitored daily by the researchers, and the veterinarian doctor visited the animals to monitor the health weekly once till the experimental procedures. Later they were safely disposed to animal ware houses safely. All experimental procedures were performed according to relevant guidelines and regulations. Each human study was reviewed and approved by the Institution Ethics Committee of Affiliated Stomatology Hospital of Guangzhou Medical University.

Following the ARRIVE guidelines and regulations approved by the Guangdong Laboratory Animals Monitoring Institute (IACUC201806).

### Acquisition of Cone-beam computed tomography (CBCT) images

CBCT was scanned for 24 sec with NewTom VG (Newtom, Italy) at Affiliated Stomatological Hospital of Guangzhou Medical University under 110 kV, 5 mA, and 3.6 seconds exposure time. The field of view was 100 mm in width and 100 mm in height. The original scanned data were analyzed through the image analysis module of the QR-NNT version 2.17 software (J. Morita Manufacturing Corp., Kyoto, Japan). The measurements of alveolar bone loss were taken from the cementoenamel junction (CEJ) to the alveolar bone crest (ABC) in the interdental region at six sites ranging from the disto-palatal or distobuccal groove of the second premolar to the second molar palatal or buccal cusp. The percentage of bone loss volume from CEJ to the root apex was defined as assessing the bone height changes. The percentage of bone loss (%) = bone loss (mm) length of CEJ to root apex (mm) [26].

### Histological analysis

Decalcified paraffin-embedded gingival tissue sections of 4 μm thickness from middle-aged humans and monkeys were stained by H&E staining and immunostaining for CD34, which is expressed throughout the inflammation and repair conditions [27, 28] and CD45, as a pan-leukocyte marker, which is widely used to evaluate the inflammatory response [29]. Histological images were captured (DMLS; Leica) and analyzed by Image-J software (NIH, Bethesda, MD, USA). The number of inflammatory cells and microvessel density (MVD) in four-unit squares (50 μm × 50 μm) of periodontal connective tissue was counted for H&E staining. For IHC staining, Primary anti-CD34 (Boster, China) and anti-CD45 (Boster, China) were used and detected by secondary Anti-rabbit lgG (ZASGB, China) followed by DAB substrate staining (PerkinElmer, Waltham, Massachusetts). The quantitative analysis of the number of positive cells was determined by counting the number of stained cells in the resorption areas under a microscope at an objective magnification of 40 × and then averaged. At least four samples in each group were used for analysis.

### Characterization of the oral microbiome using 16S rRNA genes

Unstimulated human (n = 5) and animal saliva samples (n = 4) were collected between 8:00–1:00 am according to the manufacturer’s instructions (Salivette, Germany). After collection,the saliva samples were stored at 4°C for 15 min after removal of cellular and food debris that is after centrifugation at 1500 rpm and the analysis was done immediately. However some samples were stored at -20°C for 15 days and no preservatives were added. The genomic DNA of each sample was extracted using the fecal genomic DNA extraction kit (Tiangen Biotech CO., LTD, China) according to the manufacturer’s preparation protocol.

The quality control of extracted DNA was measured using a NanoDrop ND-1000 spectrophotometer(Thermo Fisher Scientific, Waltham, MA) and agarose gel electrophoresis, respectively. The results showed that the A_260_/A_280_ ratios were all between 1.8 and 2.0 and that the DNA concentrations were 20–150 ng/μL, indicating that the extracted genomic DNA was ideal and met the requirements for subsequent sequencing. Extracted DNA (20 ng) from each sample was PCR amplified according to the TruSeq Nano DNA LT Library Prep Kit for library preparation. The 16S rDNA V4 area-specific primer 520F (5’-barcode + AYTGGGYDTAAAGNG-3’) 802R (5’-TACNVGGGTATCTAATCC-3’) were used to amplify the V4 region of 16S ribosomal DNA. High throughput sequencing was performed by Shanghai Personal Biotechnology Co., Ltd with MiSeq Reagent Kit V3 (600cycles) paired-end runs. Sequences with ≥ 97% similarity were assigned to the same operational taxonomic units (OTUs) by Vsearch (v2.3.4). Representative sequences were chosen for each OTU, and taxonomic data was then assigned to each representative sequence using the RDP (Ribosomal Database Project) classifier. The dominant species’ differences in different groups and multiple sequence alignment were conducted using the Mafft software (V 7.310) to study the phylogenetic relationship of different OTUs. Alpha diversity is applied in analyzing the complexity of species diversity for a sample through 5 indices, including Chao1, Observed species, Goods coverage, Shannon, and Simpson. All these indices in our samples were calculated with QIIME (Version 1.8.0). Beta diversity analysis was used to evaluate the differences of samples in species complexity and was was calculated by PCoA and cluster analysis using QIIME software (Version 1.8.0).

### Statistical analysis

CBCT alveolar bone loss of humans and monkeys were calculated in percentages and the mean percentages with standard deviations were derived. The student t test was performed to know the statistical difference and it was found as p<0.05. The humans and monkeys were examined for 6 phyla and the predominant species were expressed in percentages. The comparison between two groups was assessed using students t test. Different indices for oral microbiome such as alpha diversity index, Chaos -1 and Shannon index were used to know the predominant oral microbiome in humans and monkeys. The microbial analysis was done using stacked bar graph which illustrate the abundances of phyla and genera. HS: human saliva, MS- Monkey saliva. The relative distribution of sequences in the OTUs of the nine oral samples at the phylum and 225 genus levels were estimated. A value of p < 0.05 was considered to be statistically significant.

## Results

### Alveolar bone resorption was higher in humans compared with monkeys

**Fig 1:**
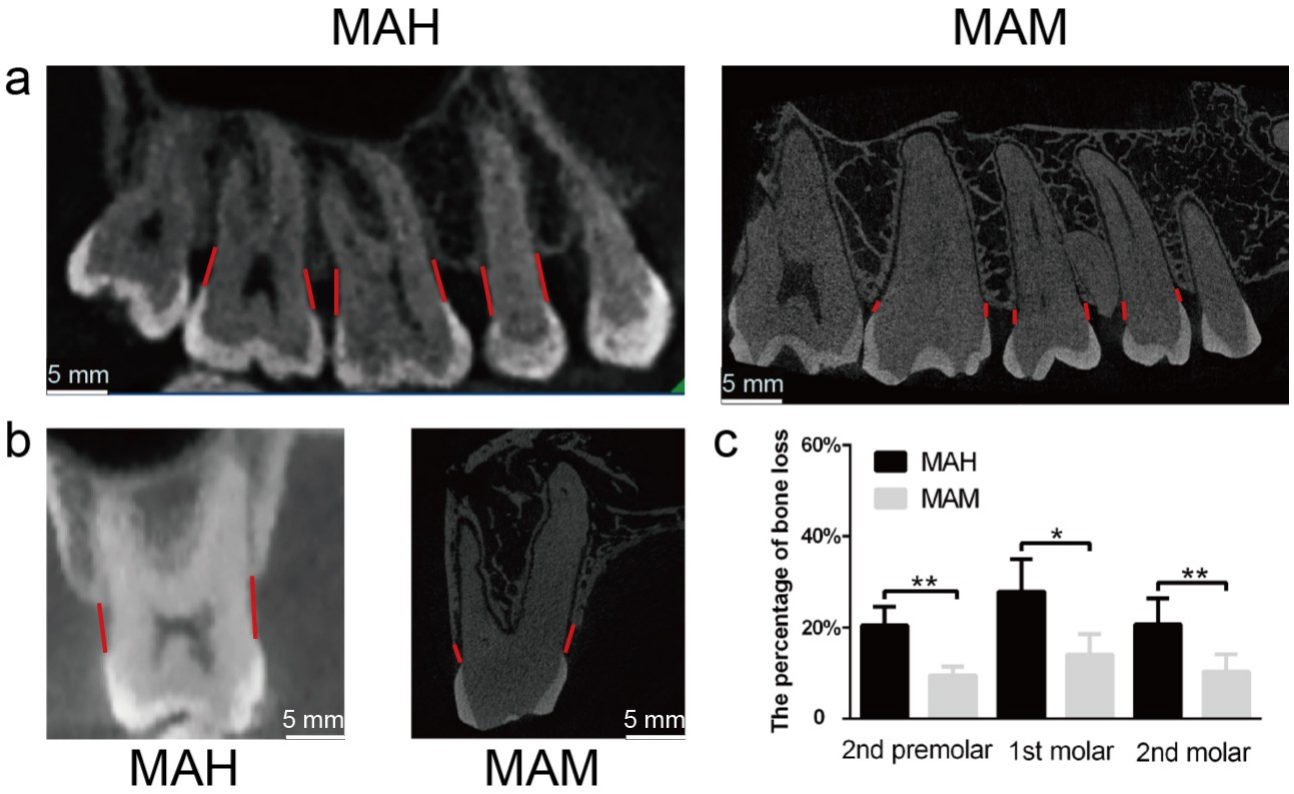
Represents the alveolar bone resorption in both the groups.

The alveolar bone level is an important sign of periodontal health. Fig 1:Higher alveolar bone resorption was observed in humans compared with monkeys. (a) The representative sagittal images show the height of the alveolar bone in the second premolar, first molar, and second molars. There was decreased height of alveolar crest 4.3mm from the level of CEJ (Cementoenamel Junction) irt to premolars where as it was 2.6mm in molars in human samples, where as it was 1.7mm in premolars and molars in monkeys as measured in sagittal section of CBCT. Hence the bone loss was more in humans when compared to monkeys. As shown in Fig 1, the alveolar bone around the posterior teeth in humans was observed. On the contrary, minor bone resorption was seen surrounding the posterior teeth in monkey. Quantitative analysis of the CBCT(Cone beam computed tomography)results demonstrated that the percentage of posterior teeth’s alveolar bone loss in humans ranged from 20.3 (± 2.8%) to 28.8 (± 5.2%). In comparison, monkeys’ results showed lower values ranging from 6.7 (± 1.6%) to 13.8 (± 3%). Thus, it is very evident that the percentage of attachment loss around posterior teeth was significantly higher in humans than in monkeys (P < 0.05), (Fig 1c). In addition to that, the result also indicated that more severe reduction in maxillary posterior alveolar average bone height in humans compared with monkeys.

Higher alveolar bone resorption was observed in humans compared with monkeys. (a) The representative sagittal images show the height of the alveolar bone in the second premolar, first molar, and second molars. (b) The representative coronal images show both groups’ alveolar bone loss around the first molar. (c) Quantitative analysis of alveolar bone loss around the first molar using CBCT data. Data are presented as mean ± SD. Statistical significance between the group, *P < 0.05 and **P < 0.01. Scale bar: 5 mm. MAH: middle-aged human, MAM: middle-aged monkey. The data can be seen in Fig 1

### A higher degree of gingival inflammation was observed in humans than in monkeys

**Fig 2:**
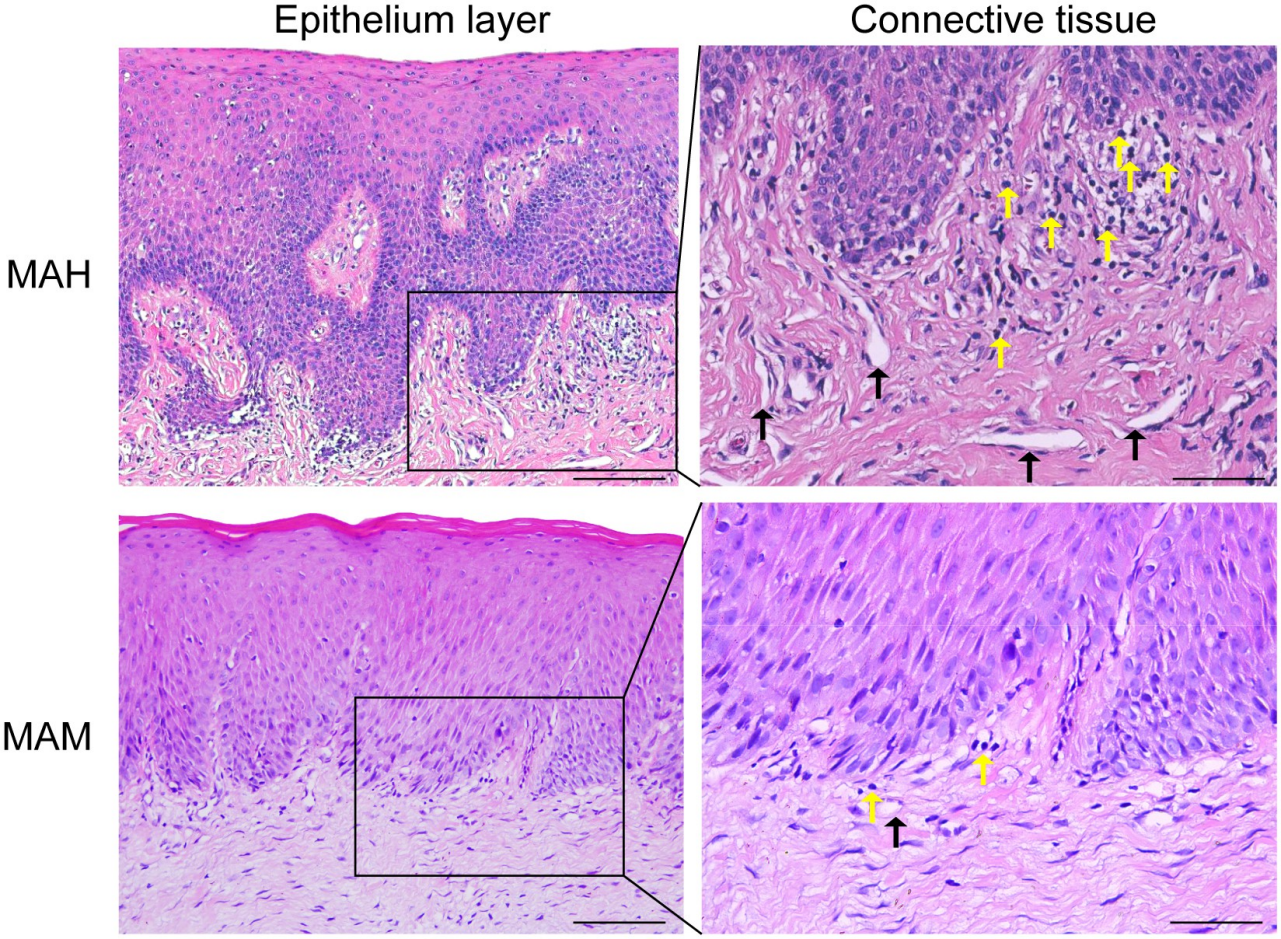
Histological images of H&E stained gingival tissue sections. The black arrow indicates blood vessels, while the yellow arrow indicates inflammatory cells. Scale bar: 100 μm. MAH: middle-aged human, MAM: middle-aged monkey.

**Fig 3:**
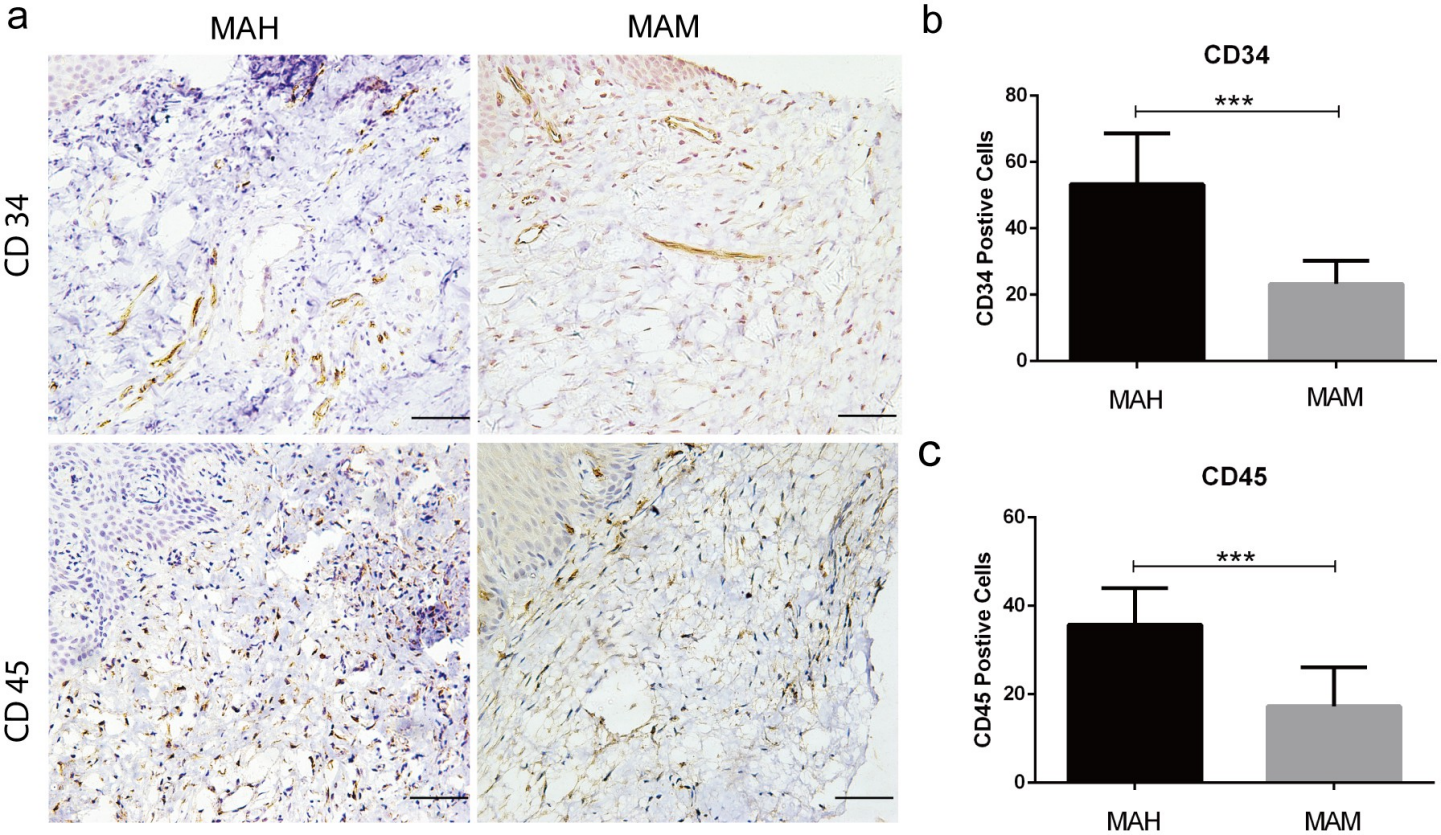
The expression of CD 34 and CD 45 in both groups.

Histological analysis was performed to visualize and compare the inflammatory changes within the gingival tissue of humans and monkeys. Firstly, the gingival sections of the human displayed significantly altered structures: (a) epithelium appears acanthosis (enlargement of spinocellular layer) and atrophy, and (b) infiltration of inflammatory cells in connective tissue. However, while analyzing microscopic aspects of the gingival sections of the monkey, histological examination of 16S rRNA gene gingival samples illustrated healthy aspects of epithelium and connective tissue. Besides, there was a marked change in lamina propria with higher number of inflammatory cells and blood vessels observed in the gingival tissue of humans compared with monkeys (Fig 2). To further confirm the inflammatory status, the expression of CD34 and CD45 in gingival tissues was studied thoroughly. As anticipated, the numbers of CD34-positive and CD45-positive cells in the humans were significantly higher than in the monkey (Fig 3a). In addition, immunohistochemical staining revealed more neovascular in the gingival tissues of humans compared with the monkey (Fig 3a), marked by a dramatic increase in MVD and lymphocyte infiltration (Fig 3b). Overall, results from histological analysis emphasized that inflammation was more prominent and severe in the human than in the monkey, in addition to significantly advanced alveolar bone loss and increased blood vessels.

TheHistological images of H&E stained gingival tissue sections can be seen in Fig 2. The black arrow indicates blood vessels, while the yellow arrow indicates inflammatory cells. Scale bar: 100 μm. MAH: middle-aged human, MAM: middle-aged monkey.

The expression of CD 34 and CD 45 in both groups as shown in Fig 3. (a) Representative images of CD34 and CD45 immunostained gingival tissue sections. (b) Quantitative analysis of CD34 and CD45 expression in gingival tissue section using an image from Fig 3a. Data are presented as mean±SD. The difference between the groups was statistically significant (***p < 0.001). Scale bar: 50 μm. MAH: middle-aged human, MAM: middle-aged monkey.

### Human and monkey salivary microbiome profile

**Fig 4:**
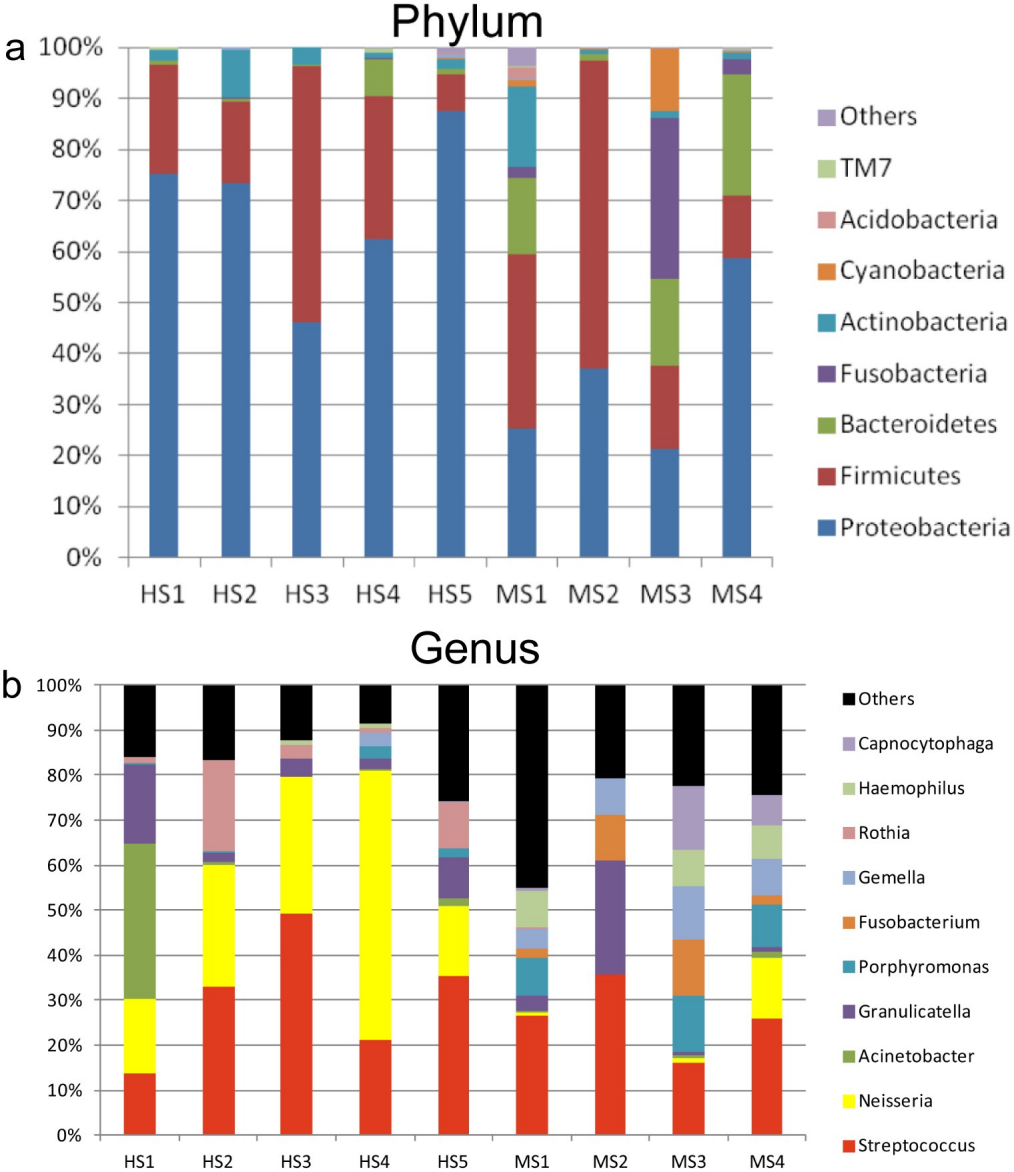
Relative distribution of sequences in the OTUs of the eight oral samples at the phylum and nine at the genus levels.

The relative distribution of sequences in the OTUs of the eight oral samples at the phylum and nine at the genus levels. Stacked bar graphs illustrate the abundances of phyla and genera. HS: human saliva, MS: monkey saliva. Eight known phyla were represented among the total OTUs along with other unknown phyla. Out of which, five phyla namely *Proteobacteria*, *Firmicutes*, *Bacteroidetes*, *Actinobacteria*, and *Fusobacteria* exhibited increased abundance greater than 1% in both salivary samples of the humans and Monkeys (Fig 4). Both human and monkey salivary microbiome had predominant *Firmicutes* and *Proteobacteria* albeit with different abundances. There was a higher abundance of *Proteobacteria* than *Bacteroidetes* in humans, while monkeys showed a reverse trend with *Bacteroidetes* (P < 0.05). At genus level, there were nine bacterial taxa with >1% abundance in human salivary samples, which included *Streptococcus* (26.5%), *Neisseria* (26.8%), *Acinetobacter* (11.4%), *Rothia* (5.2%), *Granulicatella* (3.2%), *Yersinia* (2.68%), *Porphyromonas* (1.7%), *Gemella* (1.6%), *Prevotella* (1.1%), genus level whereas twelve bacteria taxa showed >1% abundance in monkey salivary samples with highest observed in *Streptococcus* (21.1%), followed by *Porphyromonas* (10.1%), *Neisseria* (8.3%), *Fusobacterium* (7.7%), *Granulicatella* (7.6%), *Gemella* (6.1%), *Capnocytophaga* (4.3%), *Haemophilus* (4.2%), *Alloprevotella* (2.7%), *Aggregatibacter* (2.6%), *Leptotrichia* (2.1%), *Bacillus* (1.1%) (Fig 4). The microbial profiles between humans and monkeys can be differentiated by abundance of bacterial genera such as *Alloprevotella*, *Aggregatibacter*, *and streptococcus*, which is significantly high in humans compared to monkeys. Likewise Hemophilus and TM7 was abundantly seen in monkeys than humans. The relative distribution of sequences in the OTUs of the nine oral samples at the phylum and 225 genus levels were estimated in total.

**Fig 5:**
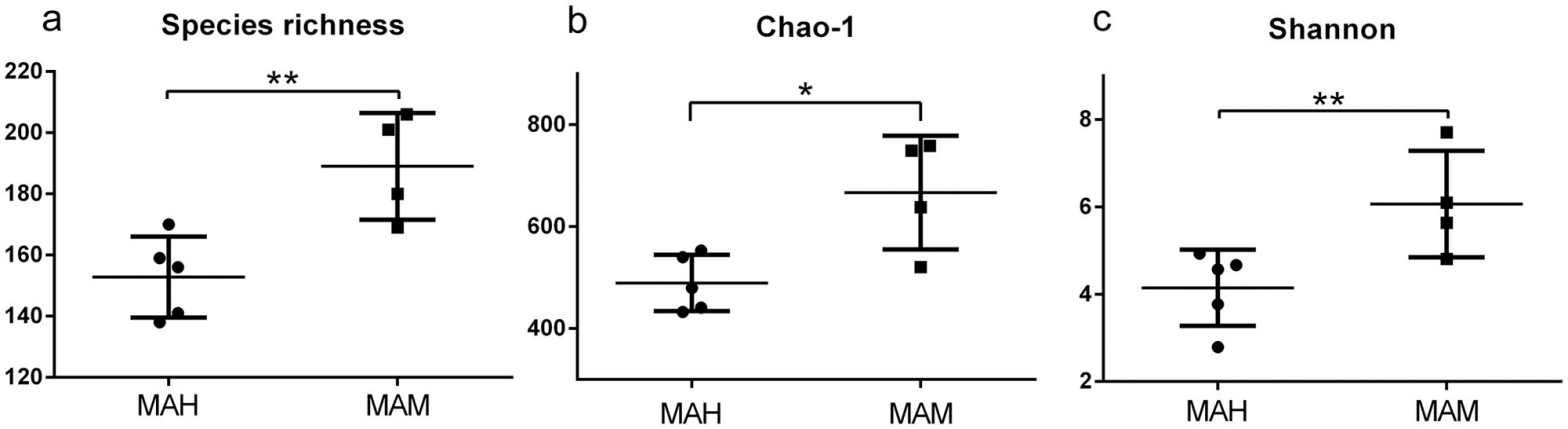
Alpha diversity index of the oral microbiome. (a) observed species richness, (b) Chao-1 index, and (c) Shannon diversity index.

The diversity, evenness, and means of each value in humans and monkeys Alpha diversity index of the oral microbiome. (a) observed species richness, (b) Chao-1 index, and (c) Shannon diversity index. Significant difference between the groups, *p < 0.05 and **p < 0.01. MAH: middle-aged human, MAM: middle-aged monkey are represented in Fig 5. Based on the scores from observed species richness (Fig 5a), Chao-1 index (Fig 5b), and Shannon diversity index (Fig 5c), the oral microbiome of *Monkey* was superior than that of humans (P < 0.05). The species richness is a measure of diversity that indicates number of different species present in given area or ecosystem. Chao- I is a measure of alpha diversity which includes species diversity within a single community. On the other hand, the alpha diversity indices revealed that the *Monkey* oral microbiota was more diverse than that in humans. It is generally accepted that a diverse community represents a stable and healthy ecosystem [30, 31]. This diversity may result from subverting defenses that would limit community composition to non-pathogenic commensals.

**Fig 6:**
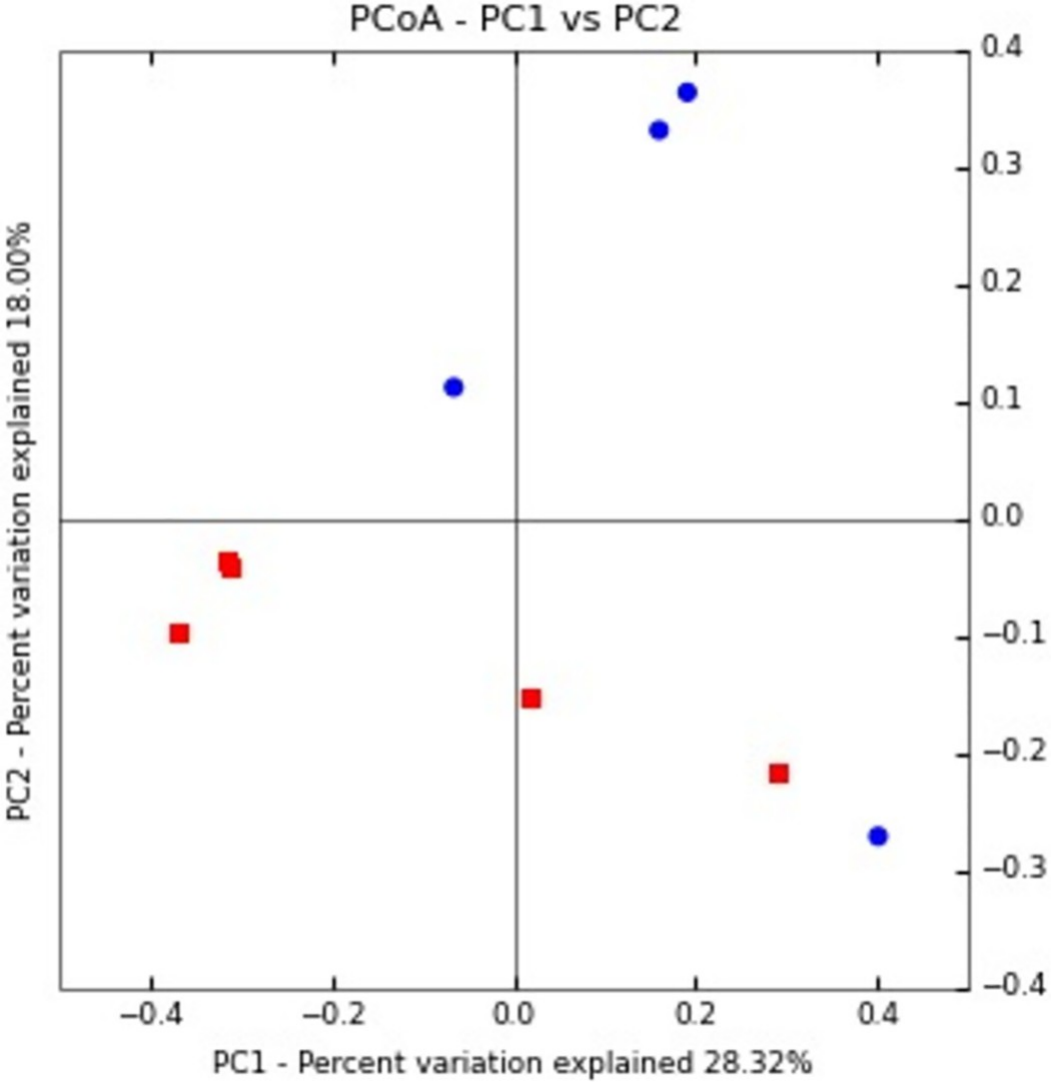
Microbiota separation on the principal coordinates calculated from unweighted UniFrac distances.

The difference between the salivary microbial profiles of humans and monkeys was evident as determined by PCoA (Fig 6). UniFrac analysis showed a significant difference in the community structure between samples. Furthermore, the human and monkey microbiome formed two distinct, separate clusters and the human oral microbiome indicated lower intra-sample variability compared with the monkey oral microbiome.

The Microbiota separation on the principal coordinates was calculated from unweighted UniFrac distances. The oral microbiome of humans and monkeys tended to cluster separately, and the human oral microbiome indicated lower intra-sample variability in comparison with that of monkeys. Red dots indicate samples from humans while blue dots indicate samples from monkeys.as shown in Fig 6.

## Discussion

The CBCT analysis was done to compare the alveolar bone resorption in humans and monkeys. The Percentage of attachment loss was more evident around the posterior teethin humans than monkeys, also the posterior alveolar bone height was greater in humans Based on CBCT results, humans displayed severe bone resorption when compared with monkeys. Even though they were middle-aged, the alveolar bone of monkeys was surprisingly found to be in a healthy state, without significant resorption. In reality, human displayed substantial bone loss, which is also observed by other studies described with similar observations [2, 32]. One of the possible explanations is the diverse oral microbiota profile driven by the different lifestyles of these subjects, which could be confounding factors for damage to periodontal tissues and account for the differences seen between humans and monkeys [33]. The next parameter we assessed was histological analysis to determine the gingival inflammation in humans and monkeys through expression of CD34, 45 in gingival tissues, we could evidently see more inflammatory changes in humans. Nonetheless, results from histological analysis also showed a higher number of inflammatory cells in the gingival tissue of humans than that of monkeys, which complements the data obtained from histological and radiological investigations. The alterations of local bacteria supragingivally and subgingivally is due to differences in the periodontal health, which reflects the salivary microbiota [14, 31, 34]. On the other hand we determine Six Phyla as operational taxonomic units where five phyla were found both in salivary samples of humans and monkeys. At the genus level total of nine bacterial taxa in human salivary samples and 12 bacterial taxa in monkey samples showed abundance of *Streptococcus* in both, with abundance of *Niesseria* in humans, however TM7 was significantly lower in monkeys thyan humans. It is likely that a core microbiota in the saliva of healthy humans exist, and plays a protective role in the health of the oral cavity [35]. In an attempt to search for possible relationships of the salivary microbiome with the above interesting results, oral saliva samples were randomly collected from middle-aged humans and monkeys, and an analysis of the microbial profile was done based on 16s RNA gene sequences.

At the phylum level, humans showed a higher abundance of *Proteobacteria* and a lower abundance of *Bacteroidetes* compared with the monkey. Differential oxygen levels might be a physical driving factor shaping the oral habitats represented by the salivary microbiome in humans and monkeys, as reported by Philippot et al [36]. At the genus level, compared with monkeys, results from humans showed a higher abundance of *TM7* and a lower abundance of *Aggregatibacter*, *Haemophilus*, *Gemella*, and *Porphyromonas*. Surprisingly, the relatively low abundance of *Porphyromonas*, *Fusobacterium*, *Aggregatibacter*, and *Haemophilus*, which are considered disease-associated species, found in human samples, despite being previously implicated in the diseased individuals [37–41]. The possible reason is the high inter-individual variability in the abundance of these samples, and due to small sample size [42] *TM7* is a novel candidate bacterial division with no cultivated representatives. Previous studies have shown microbes from this division are commonly found in the human oral flora but at relatively low abundance, with just 1% of the population, which is highly similar to the present study. Despite that, the *TM7* division was statistically enriched in human samples. A study by Liu et al indicated that this division correlated with periodontal disease, is not yet investigated much [43]. Also, other researchers suggested that the presence of a few uncultivable species, such as *TM7*, could be highly important for the manifestation of oral diseases, particularly periodontal disease [30, 43, 44] Additionally, we observed high numbers of *Neisseria* sp. in the human samples, which is in accordance with the periodontal disease samples as reported previously in the other studies [45, 46]. Above all, we speculated that, *Proteobacteria* phyla, *TM7*, might closely be associated with periodontal tissue destruction.

Dietary patterns have been shown to have a substantial impact on the salivary microbiota [35, 47, 48]. Likewise, several studies have documented an impact of diet on salivary microbiota [39, 46, 48]. The monkeys in our study were primarily frugivorous, consuming a lot of homemade feed, the main ingredients of which were similar to the vegans’ diet, while humans were generally omnivorous, but their nutrition varies individually [48]. The diversity index namely Chao-1 index and Shannon diversity index was determined to observe the species richness, where the oral microbiota of monkeys was more diverse representing health ecosystem. Furthermore, the salivary microbial composition and diversity of humans differed significantly from that of monkeys. We, therefore, speculated that different salivary microbial profiles of humans and monkeys resulted from the dietary factors, which was in accordance with the previous study (specific dietary components influence the salivary microbial community [47]. Current results are consistent with other studies looking at how dietary factors present as the primary influence on salivary bacteria. Similarly, a trans-ethnic study comparing the salivary microbiota of 52 South Koreans to that of 88 Japanese showed that the salivary microbiota of Koreans was less diverse than that of Japanese individuals, and differences in the Korean and Japanese diets were regarded as a likely factor with differences in diet [15]. Tian et al indicated that a carbohydrate-rich diet was responsible for the abundant aciduric and acidogenic salivary bacteria in human saliva [48]. Highly exposed sugar to teeth and starch consumption might be a causative factor for periodontitis along with enhanced bacteria in humans in contrast to the great apes [49] Therefore, the dietary factor may be a major factor behind the varied salivary microbial profiles, causing alternation of periodontal status. As non-human primates, cynomolgus monkeys share a similar genetic composition, oral structure, and oral microbial profile to humans [20, 50]. Interestingly, the present study indicated that periodontally healthy middle-aged humans display more severe levels of bone resorption and inflammatory infiltrate compared to healthy middle-aged monkeys. Another important finding was that the monkey oral microbiome had higher alpha diversity than humans, and different salivary profiles of these two groups were confirmed [14, 18, 34]. In view of the effect of salivary microbiota on periodontal health confirmed by other scholarsthe reasom behind the differences in the periodontal status of healthy middle-aged humans and *Monkey* was the salivary microbiome profile, which resulted from the dietary factor, although the exact reason is still unclear. In addition, the limitations of the 3 Rs (replacement, reduction, and refinement) are the most important guidelines to follow when researching experimental animal models [51]. First, non-human primates represent animal models that closely resemble humans in dental anatomy, physiology, and host immune status [22–24]. Therefore, non-human primates are the most suitable model for the current work, and the use of other species instead of primates in vitro or other non-animal alternative methodologies would not be considered. Secondly, the number of subjects included in the present study can be influenced by ethical, practical, and economic reasons. However, for the variability of individuals and better statistical power of research, reducing the number of individuals utilized is challenging to translate into clinical evidence [52]. Communication and collaboration between different laboratories could help in reducing the total number of subjects utilized. For example, the periodontal tissues of non-human primates used in the present study have been analyzed, and tissues, organs, and fluids could be preserved and used for other studies in various institutions. Adequate planning and communication between institutions could help utilize animal subjects to the fullest for better results [53]. Thirdly, to avoid or minimize pain on distress when animals are used for the sample selection, all the procedures should be performed with adequate sedation, analgesia, or anesthesia. Proper care (providing living conditions for animals) should raise more concern [51, 53].

## Conclusions

The current study is first of its kind which compared the natural periodontal status of humans and monkeys. In the present study severe bone resorption was seen in humans compared to monkeys. A lthough the etiology is still under misperception and the possibility is due to diverse oral microbiota, and different life styles of humans which can be confounding factor for damage to periodontal tissue. Moreover the TM7, which is novel candidate of bacterial division was compared in the current study, and we speculated that *Neisseria*, *Proteobacteria* might be associated with periodontal destruction. The interindividual variability in these samples may be responsible for the above findings. The dietary habits owing to carbohydrate rich diet and acidogenic salivary bacteria and teeth exposed to sugars and high starch may be causative factor in humans compared to apes. Extensive research and knowledge behind the etiology of dietary habits and risk factors for periodontal disease would effectively modulate microbial acquisition by individuals to improve long-term oral health. A more extensive series of patients and longitudinal studies are needed to confirm our findings and validate the hypothesis.

## Abbreviations

CBCT: cone-beam computed tomography
CEJ: cementoenamel junction
HE: hematoxylin-eosin staining
IHC: Immunohistochemistry
MAH: middle-aged human
MAM: middle-aged monkey
MVD: microvessel density
OTU: Operational taxonomic unit
PCoA: principal coordination analysis
US: United States
